# Supplementary material for: Compositional transformations can reasonably introduce phenotype-associated values into sparse features
Source: mSystems. 2025 May 2;10(5):e00021-25. doi: 10.1128/msystems.00021-25 (PMC12090810; doi:10.1128/msystems.00021-25)
Supplement: Supplemental Material — Fig. S1-S3. [file msystems.00021-25-s0001.pdf]

Compositional transformations can reasonably introduce phenotype-associated values into sparse features

George I. Austin[1,2], Tal Korem[2,3,†]

Author affiliations

[1] Department of Biomedical Informatics, Columbia University Irving Medical , New York, NY, USA
[2] Program for Mathematical Genomics, Department of Systems Biology, Columbia University Irving Medical Center, New York, NY, USA
[3] Department of Obstetrics and Gynecology, Columbia University Irving Medical Center, New York, NY, USA
[†] Corresponding author: tal.korem@columbia.edu

Supplementary Figures

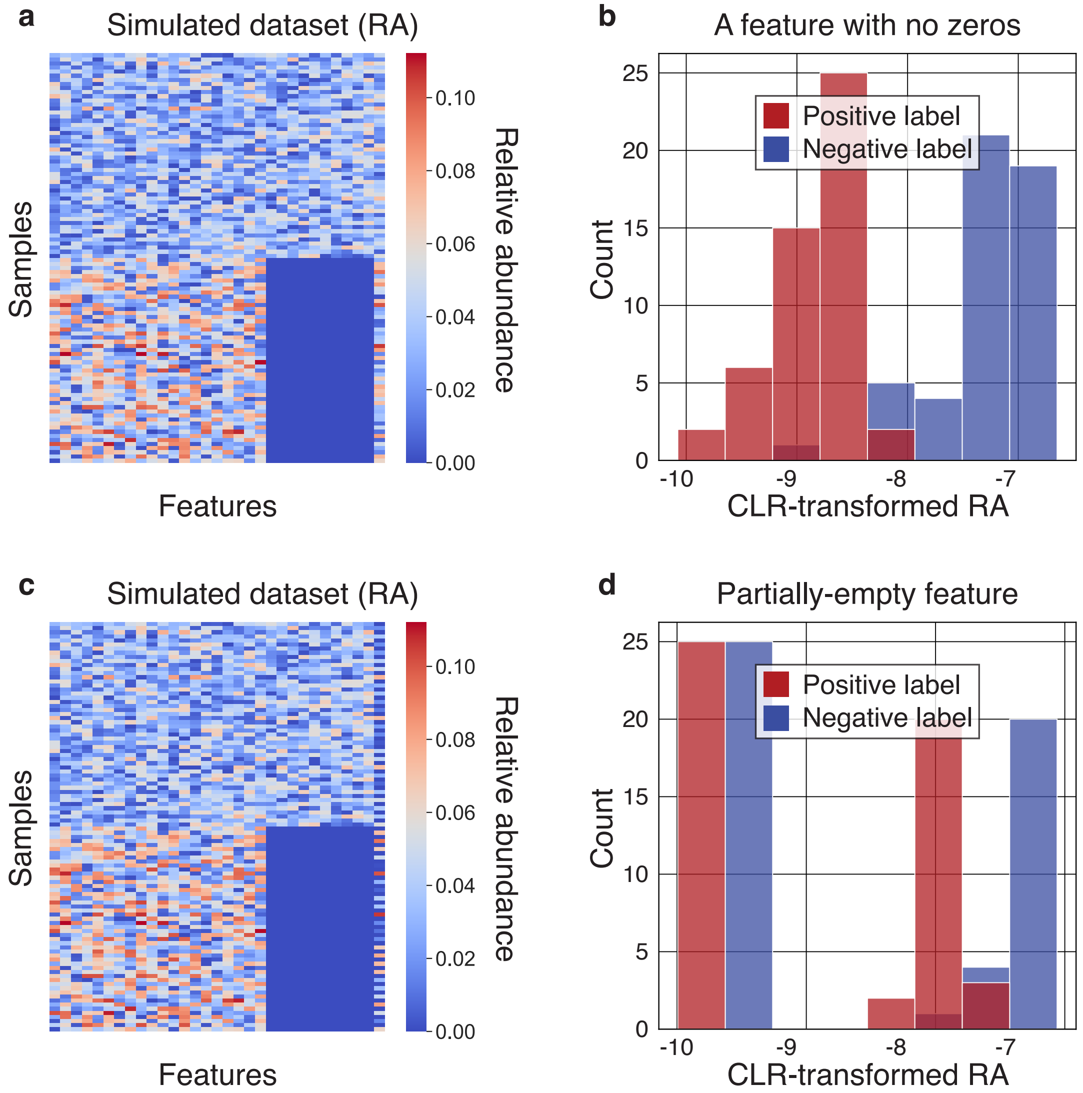

Figure S1 | Compositional transformations induce differences between groups for non-sparse and partially sparse features. a,b Same as in **Fig. 1a,d**, for a 31st feature that is drawn from the same distribution as the remaining features in "count space" before relative abundance transformation (and, in that, is equivalent to the first 20 features). **c,d** Same as in **Fig. 1a,d**, for a 31st feature that is drawn from the same distribution as the remaining features in "count space" before relative abundance transformation for 50% of samples within either group, and zeros within the remaining 50% of values.

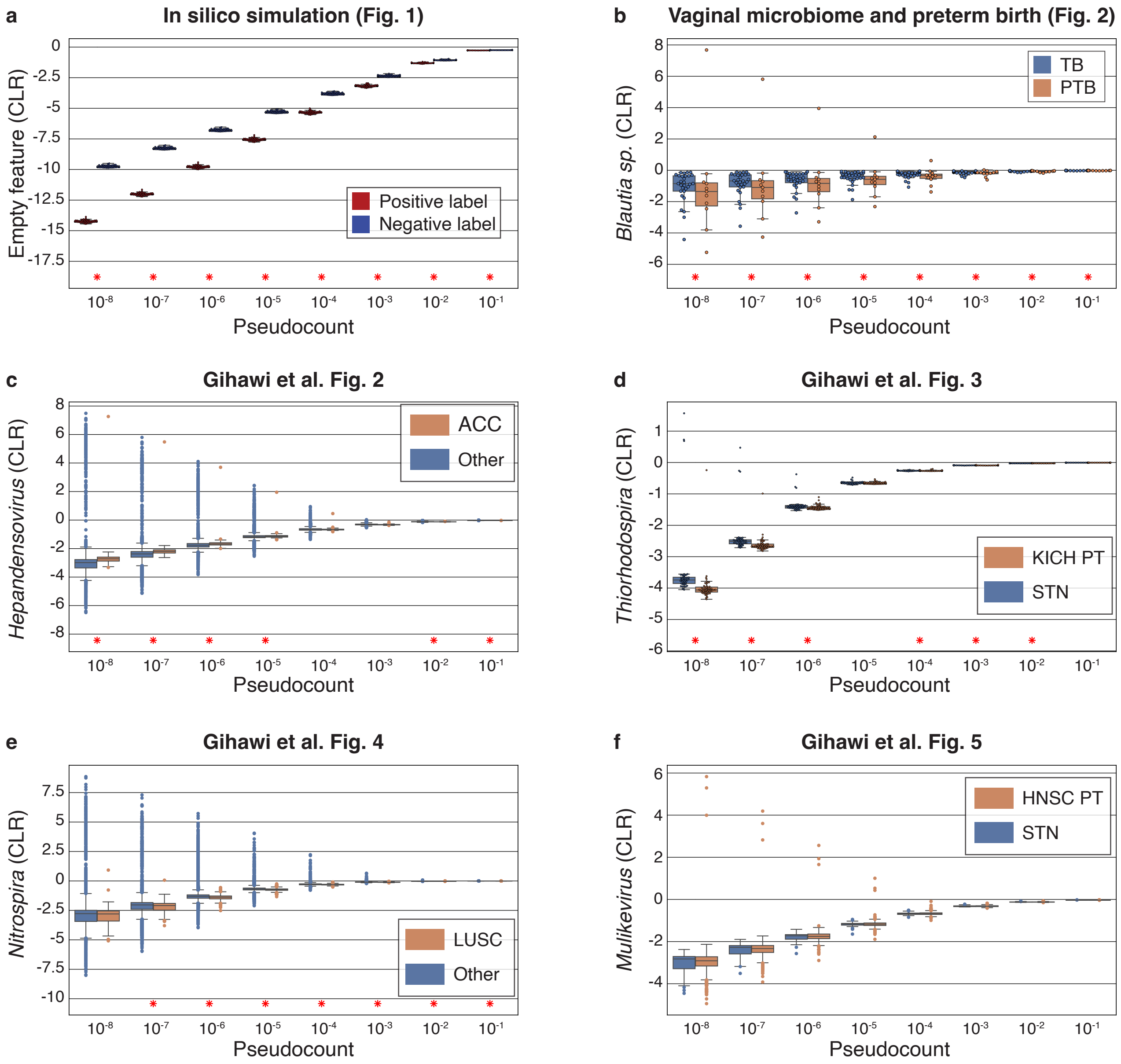

Figure S2 | Phenotype-associated values are introduced into sparse features under a range of pseudocount values. a-f, Results from the analyses performed in **Figs. 1e, 2d, and 3c,g,k,o**, but performed across a range of pseudocounts from 10^{-8} to 0.1. Box, IQR; line, median; whiskers, nearest point to 1.5*IQR. Individual dots are plotted if <= 100 samples per plot, only outliers displayed if > 100 samples. * denotes $p < 0.01$ for two-tailed Mann-Whitney U between groups.

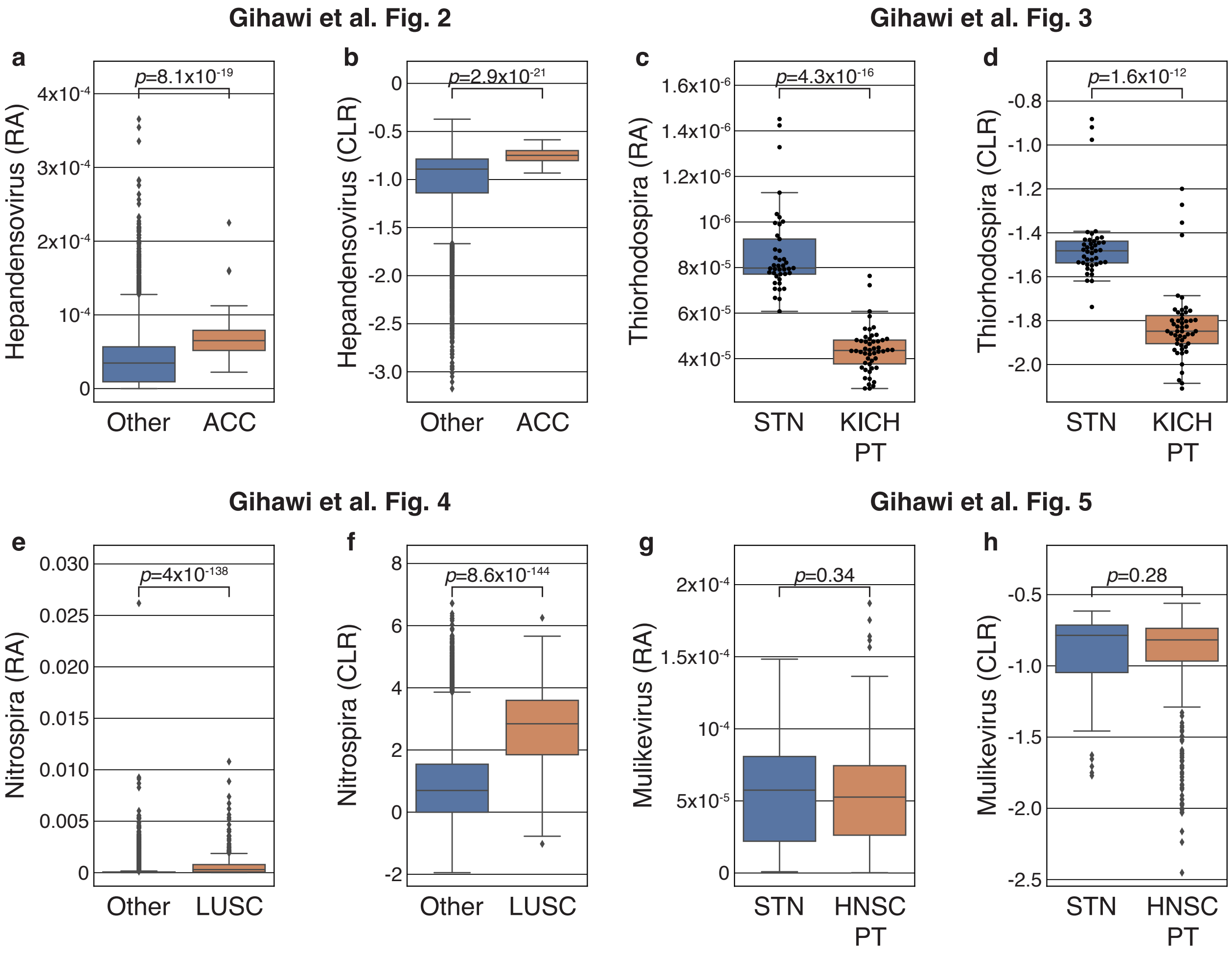

Figure S3 | An alternative pseudocount strategy increases the tumor-type associations of sparse taxa. **a,c,e,g** Boxplots similar to F**ig. 3b,f,j,n** respectively, but using a pseudocount of one in raw count space rather than introducing a pseudocount in relative abundance space. **b,d,f,h,** Boxplots similar to F**ig. 3c,g,k,o**, respectively, but using a pseudocount of one in raw count space rather than a pseudocount in relative abundance space (**Methods**). Introducing a pseudocount before normalization allows for the possibility of producing different relative abundances for sparse taxa due to differences in sample read counts, which we observe to further increase the separation across groups. While this approach is more consistent with standard Voom implementations, we have not used it in our main analyses so that all the variation induced into sparse features is explained by CLR, which is not the case here.
